# Multivessel Coronary Artery Disease Presenting as Supraventricular Tachycardia in an Elderly Woman: A Case Report

**DOI:** 10.7759/cureus.101383

**Published:** 2026-01-12

**Authors:** Abdulrahman Al-Qaysi, Zainab Al-Qaysi, Bobby Diniotis, Sarabjeet Singh, Umar Shakur

**Affiliations:** 1 Department of Family Medicine, High Street Medical Centre, Dungarvan, IRL; 2 Department of Internal Medicine, Avalon University School of Medicine, Mesa, USA; 3 Department of Cardiology, Insight Hospital and Medical Center, Chicago, USA

**Keywords:** cardiac arrythmia, coronary acute syndrome, elderly individuals, multivessel coronary disease (mvcd), supraventricular tachycardia (svt)

## Abstract

Troponin elevations occurring during supraventricular tachycardia (SVT) are often interpreted as “demand ischemia” rather than indicative of obstructive coronary artery disease (CAD). Nonetheless, differentiating transient tachycardia-related injury from an acute coronary syndrome (ACS) can be difficult. We report a case of a 71-year-old woman whose SVT episode ultimately served as the first clue to underlying severe CAD. Her progressively rising high-sensitivity troponin (HS-Tn) required further evaluation. The patient subsequently underwent coronary angiography that revealed significant multivessel coronary artery disease. This case highlights that escalating HS-Tn levels following SVT should not be automatically attributed to demand ischemia, particularly in patients with cardiovascular risk factors.

## Introduction

Globally, cardiovascular diseases (CVDs) account for the greatest burden of mortality and disability, a trend that has steadily increased over recent decades [1]. Acute coronary syndrome (ACS) is a common manifestation of CVD and is frequently complicated by tachyarrhythmias and bradyarrhythmias [2,3].

Supraventricular tachycardias (SVTs) are commonly encountered arrhythmias that can lead to meaningful morbidity and, although uncommon, may occasionally be associated with mortality [4]. Despite its higher prevalence in younger patients and women, SVT may unmask underlying ischemic heart disease in older patients with cardiovascular risk factors [5].

Several factors are thought to contribute to troponin leak in SVT, including increased heart rate, the duration of the SVT, and elevated left ventricular end-diastolic pressure. These alterations can impair coronary perfusion and lead to mild, transient ischemia [5]. It is essential, yet often difficult, to differentiate troponin rises due to SVT from those caused by ACS, since errors can postpone the detection of serious coronary pathology [5].

We present the case of an elderly woman in whom an episode of SVT led to the diagnosis of severe multivessel coronary artery disease (CAD), highlighting the importance of careful biomarker monitoring and timely diagnostic evaluation in high-risk patients.

## Case presentation

**Figure 1:**
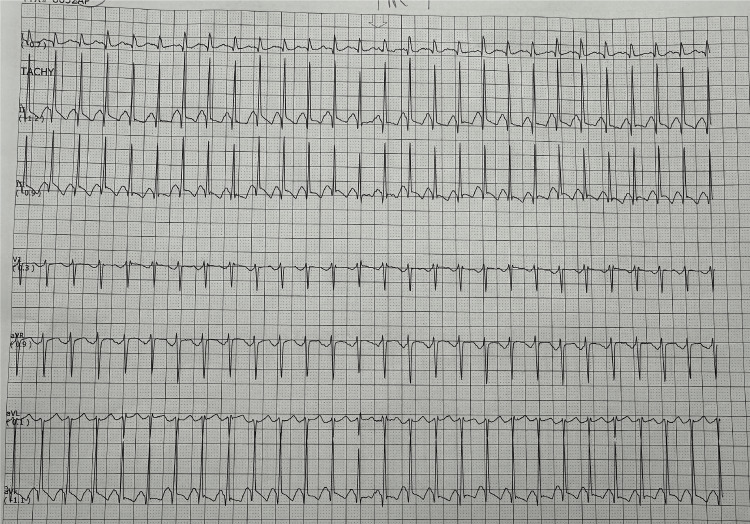
EKG shows a narrow QRS tachycardia without visible sinus P waves, consistent with SVT EKG, electrocardiogram; SVT, supraventricular tachycardia

A 71-year-old woman with a medical history of hypertension, hyperlipidemia, and type 2 diabetes mellitus presented to the emergency department (ED) following referral from her primary care physician (PCP). Earlier in the day, she visited her PCP for palpitations and was found to be hypotensive (blood pressure {BP}: 89/55 mmHg), and an electrocardiogram (EKG) showed supraventricular tachycardia (SVT) (Figure 1), prompting referral to the ED for further evaluation.

**Figure 2:**
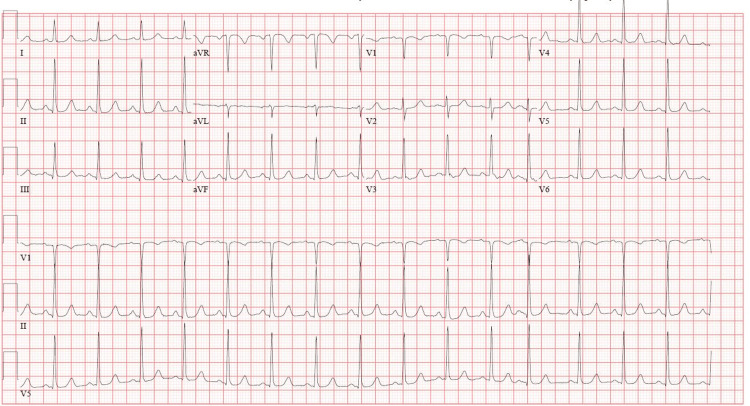
EKG at ED presentation: regular sinus rhythm with no acute changes EKG, electrocardiogram; ED, emergency department

Upon arrival, the patient denied palpitations, chest pain, dyspnea, or dizziness, and her vital signs were stable. EKG revealed sinus tachycardia without evidence of SVT, ST segment changes, or T wave inversion (Figure 2). Initial high-sensitivity troponin (HS-Tn) was 29 ng/L. The remaining laboratory results were within normal limits. She received an extra 25 mg of her home metoprolol succinate and was admitted to telemetry for monitoring.

During hospitalization, the patient remained asymptomatic. However, serial HS-Tn levels rose to 194 ng/L and later to 230 ng/L. While continuous telemetry monitoring revealed no recurrence of SVT, it showed frequent premature ventricular complexes (PVCs) (approximately one per minute). Transthoracic echocardiography demonstrated a left ventricular ejection fraction of 60%-65% with no regional wall motion or structural abnormalities.

**Figure 3:**
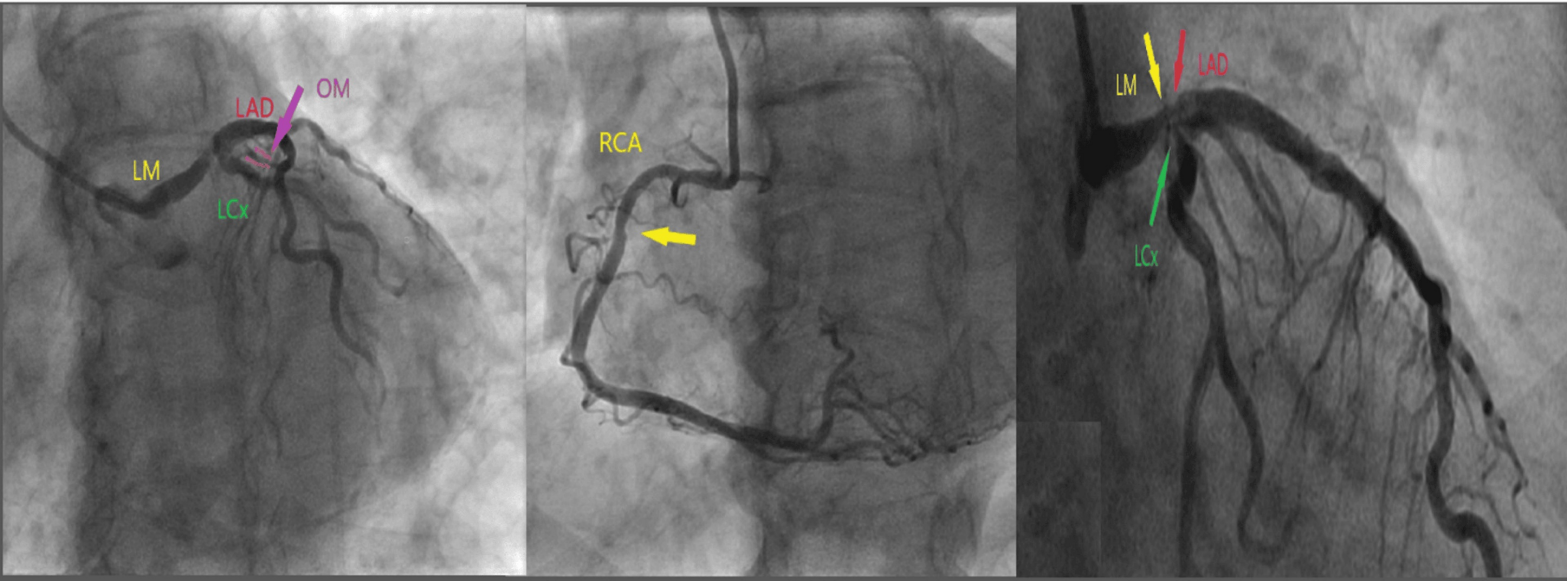
Coronary angiography revealed multivessel disease Coronary angiography shows 95% distal left main (LM) stenosis (yellow right arrow), 80% left anterior descending (LAD) stenosis with mild calcification (red arrow), 90% left circumflex (LCx) stenosis (green arrow), 90% first obtuse marginal (OM1) stenosis (purple arrow), and 70% right coronary artery (RCA) stenosis (yellow middle arrow)

Given the rising troponin levels and frequent PVCs concerning for ongoing acute coronary syndrome, coronary angiography was performed and exhibited multivessel disease, including 95% distal left main stenosis, 80% left anterior descending (LAD) stenosis with mild calcification, 90% left circumflex (LCx) stenosis, 90% first obtuse marginal (OM1) stenosis, and 70% right coronary artery (RCA) stenosis (Figure 3 and Video 1).

**Video 1:** Multivessel coronary artery disease

Heparin infusion, aspirin 81 mg daily, and high-intensity statin were initiated, and metoprolol succinate 25 mg twice daily was continued. Following this, she was transferred to a tertiary care center for evaluation for coronary artery bypass grafting (CABG).

## Discussion

Cardiovascular diseases (CVDs) continue to be the most common cause of death and disability globally, with their burden continuing to rise since 1990 due to population growth, aging, and persistent exposure to modifiable risk factors [1]. CVD is the leading cause of cardiovascular morbidity and mortality in the United States and globally and the main contributor to disability-adjusted life years (DALYs), highlighting the importance of early detection and management [1].

Coronary artery disease (CAD), a common presentation of CVD, results from atherosclerotic plaque within the coronary arteries, leading to reduced myocardial perfusion and potential progression to acute coronary syndrome (ACS) [2]. ACS typically presents with acute substernal chest pain radiating to the neck or left arm, accompanied by dyspnea, palpitations, dizziness, syncope, or acute heart failure [2]. Patients with ACS are at risk for arrhythmias, including atrial fibrillation, ventricular arrhythmias, or bradyarrhythmias, though SVT is rarely reported, making ACS diagnosis challenging when SVT coincides with elevated troponin [3].

SVTs are common cardiac arrhythmias that can cause significant morbidity and, rarely, mortality. They are often paroxysmal but may persist or lead to hemodynamic instability, typically affecting younger individuals than those with other cardiovascular diseases. Symptoms vary and may include palpitations, exercise intolerance, dyspnea, dizziness, angina, presyncope, or syncope [4].

For suspected ACS, a 12-lead EKG should be obtained within 10 minutes of presentation and HS-Tn levels measured promptly, per Class I American Heart Association recommendations. If the initial HS-Tn is nondiagnostic, repeat testing is recommended within 1-2 hours [3]. A 12-lead EKG is also the primary diagnostic tool for SVTs [4].

In patients presenting with SVT, troponin elevation may occur; however, studies suggest that this does not necessarily indicate obstructive CAD. Studies have also shown that only about 14% of patients with SVT with elevated troponin levels have clinically significant CAD [5]. These findings emphasize the value of serial troponin assessment for excluding ACS, as was performed in our patient.

In ACS, initial management includes chewable aspirin 324 mg, nitrates (if not contraindicated), early β-blockers, and high-intensity statins. Percutaneous coronary intervention (PCI) is indicated for ST elevation within the appropriate time window or for non-ST elevation myocardial infarction (NSTEMI) if risk stratification, such as the Thrombolysis in Myocardial Infarction (TIMI) score, is high [2,3]. For SVT, hemodynamically stable patients are managed first with vagal maneuvers such as carotid massage, followed by IV adenosine, calcium channel blockers, or β-blockers. Hemodynamically unstable patients require immediate direct current (DC) cardioversion [4].

## Conclusions

This case illustrates that transient SVT, along with rising troponin levels and multiple PVCs, can indicate underlying multivessel coronary artery disease even in the absence of symptoms or EKG changes. In elderly patients, transient tachyarrhythmias with elevated cardiac biomarkers should prompt evaluation for ischemia, using troponin trends, telemetry, and risk factors for timely management.
